# Supplementary material for: The dynamics of emotions in online interaction
Source: R Soc Open Sci. 2016 Aug 10;3(8):160059. doi: 10.1098/rsos.160059 (PMC5108936; doi:10.1098/rsos.160059)
Supplement: The dynamics of emotions in online interaction - Supplementary Information [file rsos160059supp1.pdf]

The dynamics of emotions under online interaction – Supplementary Information

David Garcia[1], Arvid Kappas[2], Dennis Küster[2], Frank Schweitzer[1]
[1]Chair of Systems Design, ETH Zurich, Kreuzplatz 5, 8092 Zurich, Switzerland
[2]Jacobs University Bremen, Campus Ring 1, 28759 Bremen, Germany

August 8, 2016

1 SI: Example of thread post

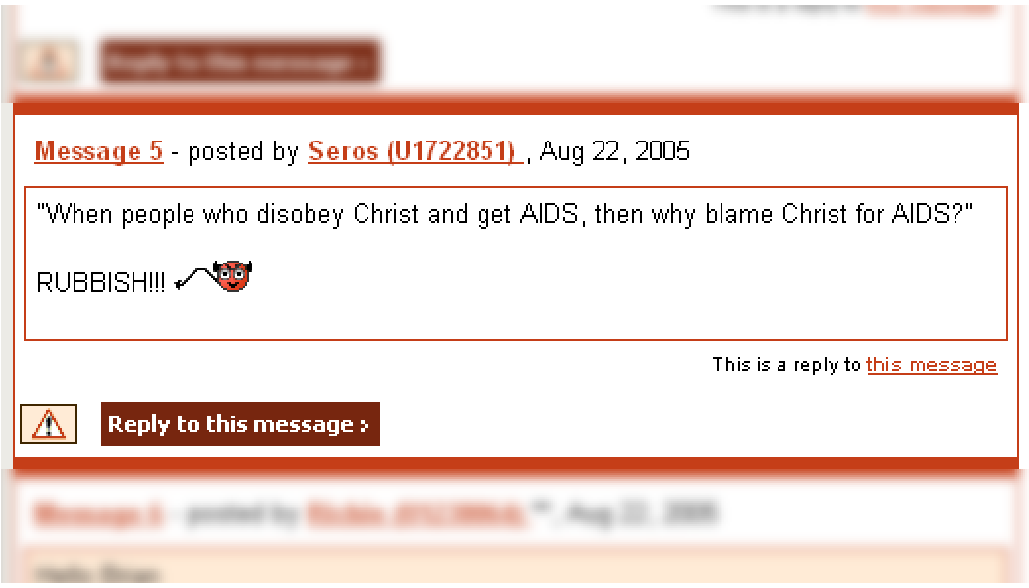

Figure 1: Example of a post from a thread used as a negative stimulus in study 1 and study 2. This thread was extracted from the BBC forum, on a topic related to religion.

2 SI: Detailed perception regression results

2.1 Valence Maximum Likelihood

The maximum likelihood method we use [1] minimizes the Akaike Information Criterion [2], defined as AIC $= 2k - 2\ln(L)$, where L is the likelihood of the model and k its amount of parameters. We test the relevance of each order in the regression by transforming the model to a linear equation of the form:

$$\frac{\Delta v(t)}{\Delta t} = I_v + c_v v(t) + b_0 h + b_1 h v(t) + b_2 h v(t)^2 + b_3 h v(t)^3 \quad (1)$$

Table 1 summarizes the full model and the most likely one, showing the decrease in AIC.

	Full Model	ML Model
I_v	$\mathbf{0.02}^{**}$	$\mathbf{0.02}^{**}$
c_v	$\mathbf{-0.37}^{***}$	$\mathbf{-0.37}^{***}$
b_0	$\mathbf{0.14}^{***}$	$\mathbf{0.14}^{***}$
b_1	0.01	
b_2	$\mathbf{0.06}^{*}$	$\mathbf{0.06}^{*}$
b_3	-0.05	$\mathbf{-0.05}^{**}$
AIC	222.17	220.19
BIC	258.20	251.08
Log Likelihood	-104.08	-104.10

$^{***}p < 0.001$, $^{**}p < 0.01$, $^{*}p < 0.05$

Table 1: Maximum Likelihood results for valence.

As a conclusion, we include in our analysis all parameters up to order 3, with the exception of b_1.

2.2 Experiment effect in valence

We test the effect of the different experiments through an interaction model with a variable E that takes a value of 0 in Study 1, and 1 in Study 2.

$$\frac{\Delta v(t)}{\Delta t} = -(\gamma_v + E\gamma_v') \left[v(t) - (b + Eb')\right] + h \left[b_0 + Eb_0' + (b_2 + Eb_2')v(t)^2 + (b_3 + Eb_3')v(t)^3\right] \quad (2)$$

This way, the value of parameters p' measure the effect of the experimental setup of Study 2 with respect to Study 1 in our analysis. The results of this interaction model are summarized in Table 2

parameter	γ_v	b	b_0	b_2	b_3
estimate	0.39***	0.055*	0.132***	0.086**	−0.079***
parameter	γ_v'	b'	b_0'	b_2'	b_3'
estimate	−0.064*	0.001	0.032	−0.074	0.070

Table 2: Parameter estimations of equation 1. $^{*}p < 0.05$, $^{**}p < 0.01$,$^{***}p < 0.001$

The estimates of all parameters of the original model are still significant and of similar values, and the experiment-dependent parameters are not significant with the exception of γ_v'. Nevertheless, the small estimate of γ_v' leads to the conclusion that there is no relevant different between studies, showing that both the laboratory and the computer setups give similar results.

2.3 Arousal Maximum Likelihood

Similarly as with valence, we test the relevance of each order in the regression by transforming the model to a linear equation of the form:

$$\frac{\Delta a(t)}{\Delta t} = I_a + c_a a(t) + d_0|h| + d_1|h|a(t) + d_2|h|a(t)^2 + d_3|h|a(t)^3 \quad (3)$$

Table 3 summarizes the full model and the most likely one, showing the decrease in AIC.

	Full Model	ML Model
I_a	$\mathbf{-0.18}^{***}$	$\mathbf{-0.18}^{***}$
c_a	$\mathbf{-0.41}^{***}$	$\mathbf{-0.41}^{***}$
d_0	$\mathbf{0.18}^{***}$	$\mathbf{0.18}^{***}$
d_1	$\mathbf{0.17}^{***}$	$\mathbf{0.14}^{***}$
d_2	-0.02	
d_3	-0.05	
AIC	153.28	150.89
BIC	189.31	176.63
Log Likelihood	-69.64	-70.44

$^{***}p < 0.001$, $^{**}p < 0.01$, $^{*}p < 0.05$

Table 3: Maximum Likelihood results for arousal.

As a conclusion, we include in our analysis all parameters up to order 1, leaving out d_2 and d_3.

2.4 Effect of signed h in arousal

We tested a possible relationship of arousal dynamics to signed dependencies on h, i.e. if it holds that arousal dynamics only depends on $|h|$. To do so, we repeated the AIC optimization method on an extended model that includes both dependences on h and $|h|$:

$$\begin{aligned} \frac{\Delta a(t)}{\Delta t} = \; & I_a + c_a a(t) + d_0|h| + d_1|h|a(t) + d_2|h|a(t)^2 + d_3|h|a(t)^3 \\ & + f_0 h + f_1 h a(t) + f_2 h a(t)^2 + f_3 h a(t)^3 \end{aligned} \tag{4}$$

	Full Model	ML Model
I_a	$\mathbf{-0.18}^{***}$	$\mathbf{-0.18}^{***}$
c_a	$\mathbf{-0.41}^{***}$	$\mathbf{-0.41}^{***}$
d_0	$\mathbf{0.18}^{***}$	$\mathbf{0.18}^{***}$
d_1	$\mathbf{0.17}^{***}$	$\mathbf{0.15}^{***}$
d_2	-0.02	
d_3	-0.05	
f_0	$\mathbf{-0.03}^{***}$	$\mathbf{-0.03}^{***}$
f_1	0.00	
f_2	0.02	
f_3	0.02	
AIC	146.71	139.92
BIC	203.34	170.81
Log Likelihood	-62.36	-63.96

$^{***}p < 0.001$, $^{**}p < 0.01$, $^{*}p < 0.05$

Table 4: Maximum Likelihood results for the extended arousal model.

Table 4 shows the best fit results of the extended model. From the parameters depending on h, only a small linear effect is present on f_0, with a magnitude much smaller that the equivalent on $|h|$. While the effect is significant, the small point estimate shows that it is not sizable and the assumption of an independence from the sign h is justified.

2.5 Experiment effect in arousal

We test the effect of the different experiments through an interaction model with a variable E that takes a value of 0 in Study 1, and 1 in Study 2.

$$\frac{\Delta a(t)}{\Delta t} = -(\gamma_a + E\gamma_a') \left[a(t) - (d + Ed')\right] + |h| \left[d_0 + Ed_0' + (d_1 + Ed_1')v(t)\right] \tag{5}$$

This way, the value of parameters p' measure the effect of the experimental setup of Study 2 with respect to Study 1 in our analysis. The results of this interaction model are summarized in Table 5

parameter	γ_a	d	d_0	d_1
estimate	0.51^{***}	-0.496^{***}	0.235^{***}	0.237^{***}
parameter	γ_a'	d'	d_0'	d_1'
estimate	-0.14	0.24	-0.111^{*}	-0.148

Table 5: Parameter estimations of equation 1. $^{*}p < 0.05$, $^{**}p < 0.01$,$^{***}p < 10^{-10}$

The estimates of all parameters of the original model are still significant and of similar values, and the experiment-dependent parameters are not significant with the exception of d_0'. The laboratory setup of Study 2 had a relative attenuation effect on the constant shift of arousal when reading emotionally charged threads, but the effect is small enough not to contradict the result of a relevant d_0 value.

3 SI: Detailed production results

3.1 Participation depending on arousal

We tested the significance of the α estimate of the MARS method by fitting a linear regressor only to values of arousal that satisfy $a(t) \geq 0$. The result is an estimate $\alpha = 0.42$ with p-value below 10^{-10} and $R^2 = 0.147$.

3.2 Experiment effect in participation dependence of arousal

We introduce a control for experimental conditions in the result of participation dependent on arousal. The results for this model are summarized in Table 6. The only difference between Study 1 and Study 2 appears for very high arousals, beyond 0.6.

term	Intercept	$h(a-0)$	$h(a-0.67)$	$h(a-0)*h(E:a-0.67)$	R^2
estimate	0.1955314	0.4766772	-0.9166260	1.0515151	0.158

Table 6: MARS model with experiment control for participation dependent on arousal

3.3 Participation depending on valence

The relationship between valence and participation tendency is shown on Figure 2. Repeating the MARS analysis as with arousal shows that the breakpoint at zero is not present, and the only important positive relationship is only present above $v = 0.5$. We also tested a possible linear dependence of the form

$$p(t) = p_0 + p_e * E + \beta v(t) + \beta_E * v(t) * E \tag{6}$$

We found that the weak relation between participation tendency and valence is experiment dependent, as shown in the linear regression results of Table 7.

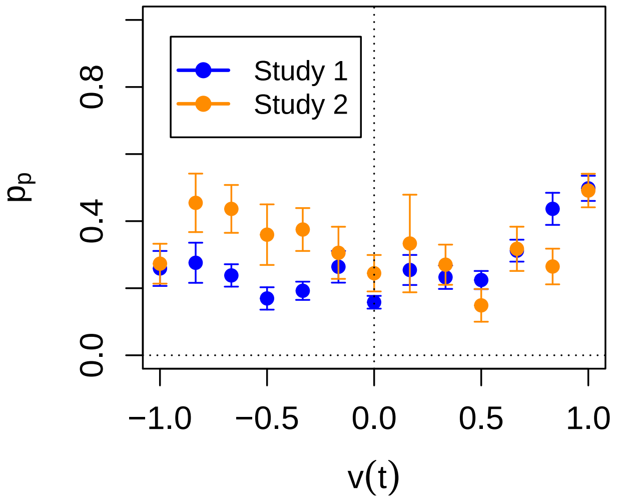

Figure 2: Mean reported participation intention given experience valence (left) in Studies 1 and 2.. Error bars show standard error. Dashed lines show MARS fits on the left, and linear regression results on the right.

parameter	p_0	p_E	β	β_E	R^2
estimate	0.25***	0.08***	0.11***	−0.10**	0.04

***$p < 0.001$, **$p < 0.01$, *$p < 0.05$

Table 7: Linear regression for participation depending on positive valence

3.4 Experiment controls in arousal feedback

We tested if the change in arousal during production depended on the previous value of the arousal, through the following model:

$$\Delta a(t) = r_0 + s * S + r_1 a(t) + r_s * S \tag{7}$$

in which variable S takes value 0 if the participant was writing a reply, and 1 if it was writing a first post. The results are shown in Table 8. The relaxation effect on arousal of writing both replies and first posts is significant, and the difference between both types of production is not significant.

	Reply	First Post	Both
r_0	-0.04	0.05	-0.04
r_1	-0.32^{***}	-0.23^{***}	-0.32^{***}
s			0.09
r_s			0.09
R^2	0.15	0.09	0.14
Adj. R^2	0.14	0.08	0.13
Num. obs.	130	130	260

$^{***}p < 0.001$, $^{**}p < 0.01$, $^{*}p < 0.05$

Table 8: Linear regression of arousal changes after production for replies, first posts, and a combined model with interaction.

3.5 Experiment controls in valence feedback

We explored the possibility of a feedback into valence after production. As shown in Figure 3, there is certain negative relation for replies.

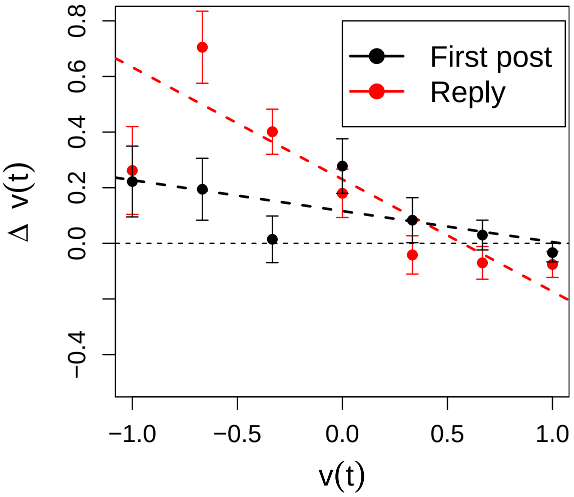

Figure 3: Change in valence when producing fist posts and comments in Study 3.

We tested the change in valence and its interaction with the type of interaction through

$$\Delta v(t) = q_0 + u * S + q_1 v(t) + q_s * S \tag{8}$$

The results are shown in Table 9, revealing the existence of a negative relation for the case of writing replies but not for first posts. This additional feedback dynamics into valence were not hypothesized in the Cyberemotions framework, and should be taken into account in future agent-based models of emotions in online interaction.

	Reply	First Post	Both
(Intercept)	0.23^{***}	0.12^{***}	0.23^{***}
	(0.04)	(0.03)	(0.04)
V	-0.40^{***}	-0.11	-0.40^{***}
	(0.07)	(0.06)	(0.07)
S			-0.11^{*}
			(0.05)
V:S			0.29^{**}
			(0.09)
R^2	0.22	0.03	0.15
Adj. R^2	0.21	0.02	0.14
Num. obs.	130	130	260

$^{***}p < 0.001$, $^{**}p < 0.01$, $^{*}p < 0.05$

Table 9: Linear regression of valence changes after production for replies, first posts, and a combined model with interaction.

3.6 Results on expression with SentiStrength

	P(pos)	P(neg)
(Intercept)	$-\mathbf{0.4203}^{*}$	0.2194
	(0.1815)	(0.1774)
v	$\mathbf{0.9462}^{**}$	$-\mathbf{0.9777}^{**}$
	(0.3006)	(0.2976)
AIC	244.7359	244.4034
BIC	251.1439	250.8114
Log Likelihood	-120.3680	-120.2017
Deviance	240.7359	240.4034
Num. obs.	182	182

$^{***}p < 0.001$, $^{**}p < 0.01$, $^{*}p < 0.05$

Table 10: Logistic regression for positive and negative expression depending on valence

	P(pos)	P(neg)
(Intercept)	−0.1110	−0.0865
	(0.1502)	(0.1502)
a	−0.2990	0.2743
	(0.2672)	(0.2670)
AIC	254.2527	255.0442
BIC	260.6607	261.4522
Log Likelihood	-125.1264	-125.5221
Deviance	250.2527	251.0442
Num. obs.	182	182

$^{***}p < 0.001$, $^{**}p < 0.01$, $^{*}p < 0.05$

Table 11: Logistic regression for positive and negative expression depending on arousal

3.7 Results on expression with QDAP

	P(pos)	P(neg)
(Intercept)	−0.1596	0.0341
	(0.1780)	(0.1721)
v	**1.0960*****	**−0.7402****
	(0.3021)	(0.2868)
AIC	240.7387	247.9446
BIC	247.1467	254.3526
Log Likelihood	-118.3693	-121.9723
Deviance	236.7387	243.9446
Num. obs.	182	182

$^{***}p < 0.001$, $^{**}p < 0.01$, $^{*}p < 0.05$

Table 12: Logistic regression for positive and negative expression depending on valence (QDAP)

	P(pos)	P(neg)
(Intercept)	0.1639	−0.2059
	(0.1502)	(0.1514)
a	−0.1305	0.3753
	(0.2662)	(0.2699)
AIC	254.9868	252.9355
BIC	261.3949	259.3435
Log Likelihood	-125.4934	-124.4677
Deviance	250.9868	248.9355
Num. obs.	182	182

$^{***}p < 0.001$, $^{**}p < 0.01$, $^{*}p < 0.05$

Table 13: Logistic regression for positive and negative expression depending on arousal (QDAP)

References

[1] Venables, W. N. & Ripley, B. D., 2002 *Modern applied statistics with S.* Springer Science & Business Media.

[2] Akaike, H., 1981 Likelihood of a model and information criteria. *Journal of econometrics* **16**, 3–14.
